# Examining the multiple mediation effects of creative personality and self-esteem on the relationship between exercise and smartphone dependence in adolescents

**DOI:** 10.3389/fpsyg.2025.1634387

**Published:** 2025-09-12

**Authors:** Jaebeom Hwang, Inwoo Kim

**Affiliations:** Department of Sports Science Convergence, College of the Arts, Dongguk University, Seoul, Republic of Korea

**Keywords:** smartphone dependence, exercise participation, creative personality, self-esteem, adolescent health

## Abstract

The widespread use of smartphones has raised growing concerns about smartphone dependence among adolescents. This study investigates how exercise participation reduces smartphone dependence by examining the mediating roles of creative personality and self-esteem. Specifically, we examined a multiple mediation model to test how these two psychological constructs function both individually and sequentially. Using secondary data from the fifth wave of the Korean Children and Youth Panel Survey 2018, we analyzed responses from 2,288 middle school students. Descriptive statistics, Pearson’s correlations, and multiple mediation analyses using bootstrapping were conducted. Results showed that greater exercise participation was associated with lower levels of smartphone dependence. While self-esteem directly mediated this relationship, creative personality influenced it indirectly through its effect on self-esteem. The sequential mediation model demonstrated that exercise participation can enhance creative personality, which in turn may increase self-esteem, potentially leading to reduced smartphone dependence. These findings highlight the psychological benefits of physical activity and suggest that promoting exercise may serve as an effective strategy for addressing adolescent smartphone dependence by strengthening self-worth and creative engagement.

## 1 Introduction

In the modern era, smartphones have become indispensable tools, offering unparalleled convenience and connectivity. However, their pervasive use has led to significant challenges, particularly among adolescents. Smartphone dependence, also known as problematic smartphone use, is characterized by compulsive behaviors that interfere with daily life and psychological well-being (Chóliz, 2010; Ding et al., 2022). According to the Korea Youth Risk Behavior Web-Based Survey, nearly 35.8% of Korean adolescents are classified as at-risk or high-risk users of smartphones, underscoring the urgency of this issue (Oh and Jung, 2021). Adolescents are especially vulnerable due to their developmental stage, which involves heightened sensitivity to social rewards and limited impulse control (Laursen and Hartl, 2013). Symptoms of smartphone dependence include excessive screen time, inability to control usage, withdrawal when access is restricted, and neglect of important responsibilities (Kardefelt-Winther, 2014). Without intervention, these patterns may lead to long-term maladaptive behaviors and psychological vulnerabilities that persist into adulthood (King et al., 2010).

Smartphone dependence has been associated with a range of negative outcomes, including diminished academic performance, reduced physical activity, and heightened psychological issues such as anxiety, depression, and loneliness (Lee et al., 2014; Pirwani and Szabo, 2024). Moreover, excessive smartphone use fosters sedentary behaviors, further exacerbating physical and mental health problems (Lepp et al., 2013; Yen et al., 2009). Given these far-reaching consequences, identifying effective strategies to address adolescent smartphone dependence has become a critical public health priority.

One promising approach to mitigating smartphone dependence is the promotion of physical activity. Numerous studies have demonstrated an inverse relationship between physical activity and problematic smartphone use (Azam et al., 2020; Xiao et al., 2022). Physical activity directly reduces sedentary behavior and improves emotional regulation, providing immediate relief from compulsive smartphone use (Tangney et al., 2018). Furthermore, regular exercise fulfills psychological needs such as autonomy, competence, and relatedness, as outlined in self-determination theory (Quested et al., 2021; Ryan, 2017). These multifaceted benefits position exercise as a cost-effective intervention for addressing adolescent smartphone dependence.

However, the mechanisms underlying the relationship between exercise and reduced smartphone dependence require further exploration. Existing research highlights the need to identify mediating variables that explain how and why physical activity reduces smartphone addiction. Investigating mediating factors such as creativity and self-esteem not only provides a deeper understanding of this relationship but also strengthens the theoretical foundation for designing targeted interventions.

Creativity and self-esteem are both positively influenced by physical activity (Bollimbala and James, 2023; Oppezzo and Schwartz, 2014; Pascoe et al., 2020; Vasilopoulos et al., 2023; Wu et al., 2024) and have been independently associated with reduced smartphone dependence (Barbot, 2020; Joinson, 2004), making them ideal candidates for mediating variables. Creativity, defined as the ability to generate novel and adaptive ideas, is significantly enhanced through regular physical activity. Mechanisms such as improved cognitive flexibility, divergent thinking, and problem-solving skills underline this effect (Bollimbala and James, 2023; Oppezzo and Schwartz, 2014). For example, fostering creativity during physical activity interventions significantly enhances cognitive and academic outcomes in children (Vasilopoulos et al., 2023). Enhanced creativity provides adolescents with alternative, meaningful outlets for engagement, reducing their reliance on smartphones for entertainment and stimulation (Bowers et al., 2014).

Similarly, physical activity has been shown to enhance self-esteem by promoting a sense of achievement, competence, and physical well-being (Pascoe et al., 2020; Wu et al., 2024). Adolescents with higher self-esteem are less likely to develop smartphone dependence, as self-esteem provides emotional stability and reduces the need to seek external validation through digital platforms (Ding et al., 2022). Self-esteem has also been shown to serve as a protective factor against problematic smartphone use (Ding et al., 2022).

Creativity and self-esteem are also interconnected, with creativity fostering confidence and self-worth. Engaging in creative activities allows adolescents to build self-esteem by affirming their abilities to solve problems and express themselves effectively (Barbot, 2020; Pérez-Fuentes et al., 2019). This synergistic relationship between creativity and self-esteem enhances their combined potential to mitigate smartphone dependence. Together, these variables amplify the protective effects of physical activity, offering a robust theoretical foundation for their inclusion in a dual mediation model.

Building on the established links among exercise, creativity, self-esteem, and smartphone dependence, the dual mediation model hypothesizes that physical activity reduces smartphone dependence through two pathways: directly by fostering creativity and self-esteem, and indirectly by leveraging the reinforcing relationship between these variables. Creativity, nurtured by physical activity, provides meaningful alternatives to smartphone use, while self-esteem acts as a buffer against dependency. Furthermore, creativity contributes to self-esteem by enhancing adolescents’ confidence in their problem-solving abilities and self-expression (Barbot, 2020; Pérez-Fuentes et al., 2019; Wang et al., 2017). This dynamic interplay creates a reinforcing cycle in which creativity and self-esteem work together to mitigate smartphone addiction.

In summary, adolescent smartphone dependence poses significant risks to physical health, mental well-being, and social development. While prior research has identified physical activity as a potential intervention, the underlying mechanisms remain insufficiently explored. This study aims to fill that gap by examining the dual mediation effects of creativity and self-esteem in the relationship between exercise participation and smartphone dependence. By empirically validating this model, we offer a more comprehensive understanding of how physical activity mitigates smartphone reliance and provide evidence-based recommendations for promoting adolescent well-being.

## 2 Materials and methods

This study utilized secondary data analysis based on the KCYPS 2018, developed and distributed by the National Youth Policy Institute. The KCYPS 2018 is a longitudinal study designed to explore the developmental trajectories and growth patterns of children and adolescents in Korea. Participants were selected through a multi-layered stratified cluster sampling process, targeting students across Korea who were in either the fourth grade of elementary school or the first grade of middle school in 2018. Stratification was conducted based on region, urbanization level, and school type, ensuring a balanced representation of students from urban, suburban, and rural areas across 17 provinces and metropolitan areas in Korea. All students within the selected classrooms were surveyed, ensuring a comprehensive and unbiased representation of adolescents nationwide.

The KCYPS dataset comprises two primary cohorts: the “fourth-grade panel,” which tracks students starting from their fourth grade of elementary school, and the “first-grade panel,” which follows students beginning from their first year of middle school in 2018. Annual data collection provides valuable longitudinal insights into various aspects of participants’ educational, emotional, and behavioral development. Both the dataset and accompanying documentation are publicly available through the National Youth Policy Institute’s website,^1^ allowing researchers to access the resources after agreeing to the terms of use.

### 2.1 Participants

This study utilized data from the fifth wave of the KCYPS 2018 fourth-grade panel. Specifically, data analyzed were collected in 2022, when participants were in their second year of middle school. Of the original 2,607 participants, the study focused on 2,288 individuals (1,141 boys and 1,147 girls) who provided complete responses to all relevant survey items.

### 2.2 Measurements

#### 2.2.1 Exercise time

Participants’ exercise time was measured using a question adapted from the International Physical Activity Questionnaire (IPAQ; Craig et al., 2003). The assessment consisted of a single-item scale asking participants to report the time spent engaging in sweat-inducing physical activity over the past week. Responses were recorded on a 5-point Likert scale, where 1 indicated “none,” 2 indicated “1 h,” 3 corresponded to “2 h,” 4 to “3 h,” and 5 represented “4 h or more.”

#### 2.2.2 Creative personality

Creative personality was assessed using data collected in the KCYPS 2018 survey. The survey employed items adapted by Choe and Pyo (2014) from the Creative Personality Scale (CPS), originally developed by Gough (1983). The adaptation by Choe and Pyo was specifically designed for individuals ranging from adolescence to middle adulthood. Their validation study confirmed the appropriateness of the scale for assessing creative personality traits among Korean adolescents, taking into account the cultural context. The CPS consists of 30 adjectives, with 18 representing creative traits and 12 reflecting non-creative traits. Participants selected adjectives they believed best described themselves. In the KCYPS 2018, each adjective associated with a creative trait was scored +1, while adjectives linked to non-creative traits were scored −1. Total scores ranged from −12 to 18, with higher scores indicating stronger creative personality traits. This study used the total scores provided by the KCYPS 2018 dataset.

#### 2.2.3 Self-esteem

Self-esteem was measured using items from the KCYPS 2018 survey adapted from Rosenberg’s Self-Esteem Scale (Rosenberg, 1965). The survey included statements such as “I have a positive attitude toward myself” and “I feel that I am a person of worth, at least on an equal plane with others.” Participants responded to 10 items using a 4-point Likert scale, ranging from 1 (“Strongly disagree”) to 4 (“Strongly agree”). Five items were negatively worded (e.g., “At times, I think I am no good at all”) and reverse-scored to ensure consistency in the scoring direction. Higher average scores indicated higher self-esteem. The reliability of the self-esteem scale, as measured by Cronbach’s alpha, was 0.812.

#### 2.2.4 Smartphone dependence

Smartphone dependence was assessed using the Short Version of the Smartphone Addiction Self-Diagnosis Scale developed by Kim et al. (2012). This instrument comprises 15 items across four dimensions: daily life disturbance (5 items), virtual world orientation (2 items), tolerance (4 items), and withdrawal symptoms (4 items). Participants rated each item on a 4-point Likert scale ranging from 1 (“Not at all true”) to 4 (“Very true”), with intermediate points labeled “Somewhat untrue” and “Somewhat true.” Sample items include “I feel restless and anxious when I do not have my smartphone” and “It would be unbearable if I were unable to use my smartphone.” Negatively worded items were reverse-coded to ensure that higher scores indicated greater smartphone dependence. The scale showed strong internal consistency in the KCYPS 2018 dataset, with a Cronbach’s alpha of 0.856.

### 2.3 Statistical analysis

Data were obtained from the National Youth Policy Institute website (see footnote 1) and analyzed using IBM SPSS Statistics (Version 26.0) and the PROCESS macro for SPSS (Hayes, 2012). Descriptive statistics, including means, standard deviation, skewness, and kurtosis, were computed to assess data distribution and examine the assumption of normality. In accordance with the guidelines of Kline’s (2015), skewness and kurtosis values were acceptable if their absolute values were ≤ 3 and ≤ 8, respectively.

Pearson’s product–moment correlation coefficients (r) were calculated to examine bivariate relationships among the study variables. Multiple mediation analysis was conducted using PROCESS Macro Model 6, which accommodates multiple mediators and allows for the assessment of both parallel and sequential mediation pathways (Hayes, 2012).

Bootstrapping with 5,000 resamples was employed to test the significance of indirect effects using 95% bias-corrected confidence intervals. An indirect effect was considered statistically significant if the confidence interval did not include zero (Preacher et al., 2007).

## 3 Results

### 3.1 Descriptive statistics

The results of the descriptive statistics analysis are summarized in Table 1. The mean score for physical activity time was 2.51, indicating that, on average, participants engaged in sweat-inducing exercise for 1 to 2 h per week. A gender difference was observed, with males (M = 2.840, SD = 1.347) reporting significantly higher physical activity time than females (M = 2.186, SD = 1.225), t = 12.151, *p* < 0.001. The creative personality score, which ranges from −12 to 18, had a mean of 0.95, suggesting a relatively low level of creative traits among the participants. In contrast, self-esteem showed a relatively high average score of 2.90. The mean score for smartphone dependence was 2.18, indicating that participants exhibited a moderate tendency toward smartphone reliance. A significant gender difference was observed, with females (M = 2.195, SD = 0.429) scoring higher than males (M = 2.174, SD = 0.455), t = −1.104, *p* < 0.05. The skewness and kurtosis values for all variables fell within the acceptable range, indicating no severe deviations from normality. This ensures that the subsequent statistical analyses, including mediation modeling, can proceed without significant concerns regarding data distribution.

**Table 1:** Descriptive statistics.

Variables	Gender	Mean	*SD*	*t*	Skewness	Kurtosis
Exercise time	Total	2.512	1.328	12.151**	0.482	−0.897
	Boys	2.840	1.347			
	Girls	2.186	1.225			
Creative personality	Total	0.950	2.697	1.665	0.539	1.332
	Boys	1.040	2.623			
	Girls	0.860	2.766			
Self-esteem	Total	2.905	0.432	1.159	0.028	0.489
	Boys	2.915	0.428			
	Girls	2.894	0.435			
Smartphone Dependence	Total	2.185	0.442	−1.104*	0.010	0.398
	Boys	2.174	0.455			
	Girls	2.195	0.429			

* Note: **p < 0.01, *p < 0.05, *N* = 2,288.

### 3.2 Correlation analysis

The correlation analysis results revealed significant relationships among all variables in the study, as shown in Table 2 (*p* < 0.01). However, the strength of these correlations was relatively weak, indicating that while associations exist, they are not particularly strong in isolation. Physical activity time was positively correlated with creative personality (r = 0.191) and self-esteem (r = 0.101), indicating that increased physical activity is associated with higher levels of creativity and self-esteem. Conversely, physical activity time was negatively correlated with smartphone dependence (r = −0.082), suggesting that greater physical activity is linked to reduced smartphone reliance. Creative personality was positively correlated with self-esteem (r = 0.269) and negatively correlated with smartphone dependence (r = −0.087). Similarly, self-esteem was negatively correlated with smartphone dependence (r = −0.279), indicating that higher self-esteem is associated with lower levels of smartphone dependence.

**Table 2:** Correlation matrix.

Variables	1	2	3	4
1. Exercise Time	1			
2. Creative Personality	0.191**	1		
3. Self-esteem	0.101**	0.269**	1	
4. Smartphone Dependence	−0.082**	−0.087**	−0.279**	1

* Note: **p < 0.01, *N* = 2,288.

While the correlation coefficients were relatively weak, the observed relationships suggest potential indirect effects that cannot be fully explained by direct associations alone. To further investigate these mechanisms, we examined whether creativity and self-esteem mediate the link between physical activity and smartphone dependence.

### 3.3 Multiple mediation effect analysis

**Figure 1:**
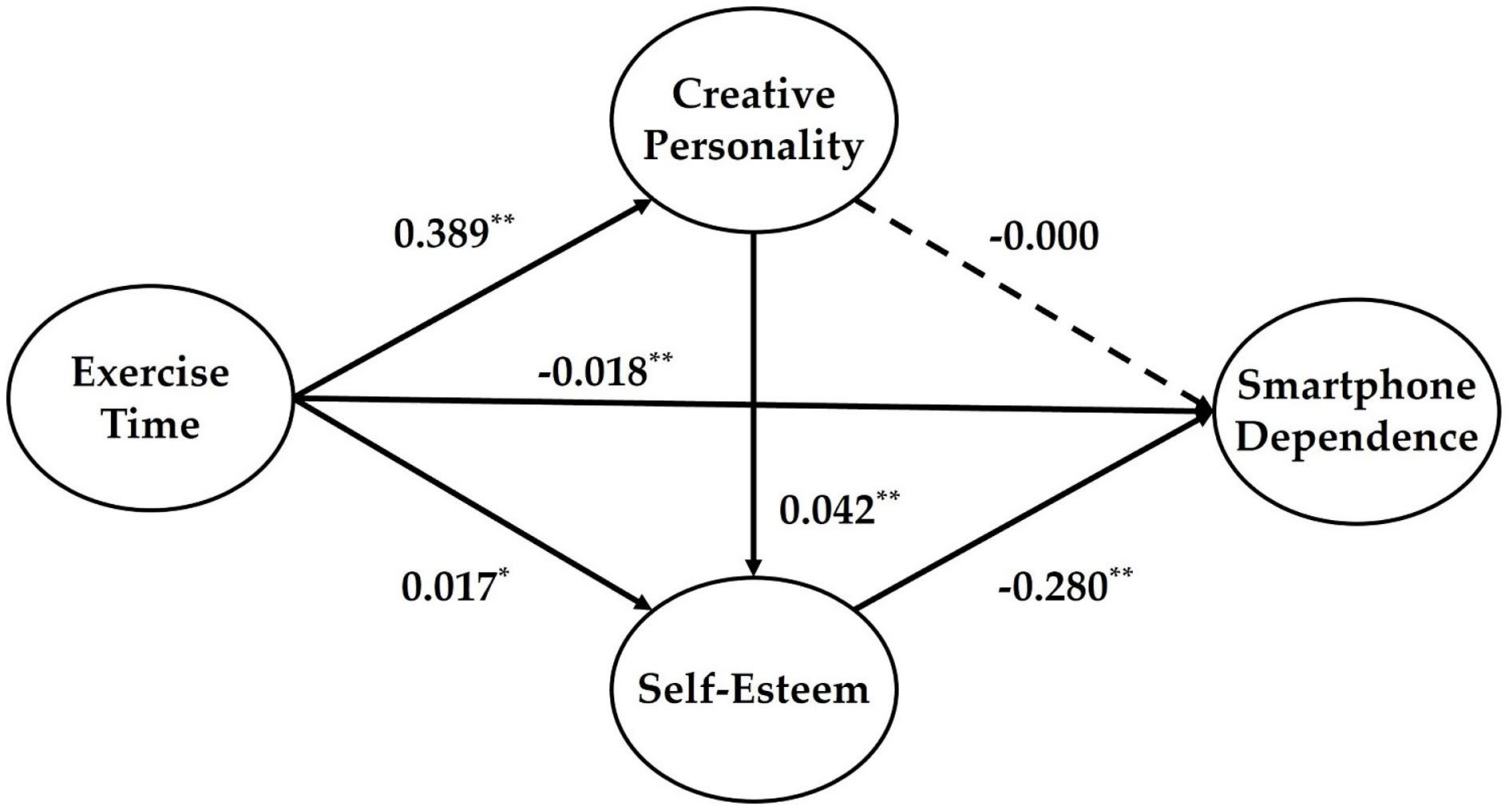
Multiple mediation model illustrating the pathways from exercise time to smartphone dependence via creative personality and self-esteem (*N* = 2,288). Solid arrows indicate significant effects (*p* < 0.01), while the dashed arrow represents a non-significant path.

To examine the multiple mediation effects of creative personality and self-esteem in the relationship between physical activity time and smartphone dependence, a regression analysis was conducted using Process macro Model 6 (Table 3). The results, summarized in Table 3 and visually represented in Figure 1, showed that the independent variable, physical activity time, positively predicted the first mediator, creative personality (B = 0.389). Additionally, physical activity time and creative personality both positively predicted the second mediator, self-esteem (B = 0.017, B = 0.042, respectively). However, while these coefficients were statistically significant, their magnitudes were relatively small, indicating that their practical effect may be modest.

**Table 3:** Regression analysis.

Independent variables	Dependent variables	*B*	SE	*ß*	*t*	*F*	*R* ^2^
Exercise Time	Creative Personality	0.389	0.042	0.191	9.320**	86.855	0.037**
Exercise Time	Self-esteem	0.017	0.007	0.051	2.498*	92.766	0.075**
Creative Personality		0.042	0.003	0.260	12.665**		
Exercise Time	Smartphone dependence	−0.018	0.007	−0.054	−2.651**	67.096	0.081**
Creative Personality		0.000	0.004	−0.003	−0.120		
Self-esteem		−0.280	0.021	−0.273	−13.098**		
Total effect		−0.027	0.007	−0.082	−3.948**	15.584	0.007**

*Note: **p < 0.01, *p < 0.05, *N* = 2,288.

In the final regression model, physical activity time (B = -0.018) and self-esteem (B = -0.280) were significant negative predictors of smartphone dependence. However, while the coefficient for creative personality (B = -0.0004) was in the negative direction, its effect was not statistically significant, indicating that creative personality did not have a meaningful direct effect on smartphone dependence in this model.

The analysis of indirect effects is summarized in Table 4. First, the indirect effect of physical activity time on smartphone dependence through creative personality was not significant (B = −0.0005, LLCI = −0.009, ULCI = 0.008). However, the mediating effect of self-esteem in the relationship between physical activity time and smartphone dependence was significant (B = −0.014, LLCI = −0.026, ULCI = −0.003). Additionally, the sequential mediation effect of creative personality and self-esteem was also significant (B = −0.014, LLCI = −0.018, ULCI = −0.010). The total indirect effect, which combines all three pathways, was significant, as the confidence interval did not include zero (B = −0.028, LLCI = −0.044, ULCI = −0.014). While the significant indirect and sequential mediation effects provide important theoretical insights, the small magnitude of the coefficients suggests that the practical impact of these pathways should be interpreted with caution.

**Table 4:** Indirect effects.

Independent variables	Path	Mediation variables	Path	Dependent variables	*ß*	SE	LLCI	ULCI
Exercise Time	→	Creative Personality	→	Smartphone Dependence	−0.001	0.004	−0.009	0.008
Exercise Time	→	Self-esteem	→	Smartphone Dependence	−0.014	0.006	−0.026	−0.003
Exercise Time	→	Creative Personality ↓ Self-esteem	→	Smartphone Dependence	−0.014	0.002	−0.018	−0.010

*Note: *N* = 2,288.

These results provide evidence for the role of self-esteem as a key mediator in the relationship between physical activity time and smartphone dependence, while also highlighting the sequential mediation pathway involving both creative personality and self-esteem. However, the individual mediation effect of creative personality alone was not statistically significant.

## 4 Discussion

The increasing prevalence of smartphone dependence among adolescents has raised serious concerns about its impact on physical and psychological health. Although physical activity has been proposed as a mitigating factor, the specific pathways through which it influences smartphone use remain insufficiently understood. This study investigated the mediating roles of creative personality and self-esteem, offering insight into the psychological mechanisms that may explain this association.

The findings suggest that engaging in physical activity may reduce smartphone dependence by lowering sedentary behavior and promoting involvement in structured, goal-directed activities. Adolescents who regularly participate in exercise tend to use smartphones less frequently, likely due to the psychological and behavioral benefits derived from physical activity. These outcomes are consistent with prior research showing that exercise supports behavioral regulation by enhancing emotional stability and reducing stress (Pascoe et al., 2020). These outcomes are also consistent with a growing body of international evidence showing similar patterns across diverse cultural contexts (e.g., Qin et al., 2025). Furthermore, physical activity satisfies essential psychological needs such as autonomy, competence, and relatedness (Quested et al., 2021; Ryan, 2017).

Beyond these direct effects, the study highlights the important mediating roles of creative personality and self-esteem. Exercise was significantly associated with the development of creative traits, likely by fostering cognitive flexibility, divergent thinking, and problem-solving abilities. While some literature suggests that creativity alone may help alleviate technology overuse (Barbot, 2020; Bowers et al., 2014), the current findings did not support a statistically significant direct effect of creative personality on smartphone dependence. Instead, its influence appears to occur indirectly through enhanced self-esteem, which in turn may play a more direct role in reducing smartphone dependence.

Self-esteem demonstrated a robust mediating role in the relationship between exercise and smartphone dependence. Participation in physical activity appears to enhance adolescents’ self-esteem through improvements in fitness, perceived competence, and opportunities for social interaction (Pascoe et al., 2020). Adolescents with stronger self-esteem may be less motivated to seek external approval through digital platforms, thereby reducing their reliance on smartphones (Ding et al., 2022; Wang et al., 2017).

The study’s most significant contribution lies in the identification of a sequential mediation pathway: exercise may enhance creative personality, which then could foster self-esteem, potentially contributing to reduced smartphone dependence. This dynamic interplay suggests that creativity and self-worth work in tandem to shape behavioral regulation. Understanding this mechanism offers valuable insights for developing interventions that address the psychological underpinnings of smartphone overuse in adolescence.

Physical activity not only reduces sedentary behavior but also provides structured opportunities for personal and social development (Wu et al., 2024). By enhancing both self-esteem and creativity—core components of adolescent emotional and cognitive growth—exercise helps equip young individuals with psychological tools to navigate the challenges of smartphone overuse (Barbot, 2020; Joinson, 2004).

Given these advantages, incorporating physical activity into school and community programs may be a practical and scalable approach to addressing smartphone dependence. Such programs can encourage adolescents to develop confidence, strengthen peer relationships, and engage in meaningful, screen-free activities. Team-based sports and creative movement initiatives, in particular, may offer viable alternatives to excessive smartphone use while supporting holistic adolescent development (Wang et al., 2017).

Despite its valuable insights, this study has several limitations that should be considered when interpreting the findings. First, while we incorporated gender differences in descriptive statistics, additional subgroup analyses were not conducted due to the homogeneous nature of the sample. The dataset comprises a longitudinal panel of middle school students who were all at the same academic level (i.e., second-year middle school), limiting the possibility of analyzing differences across age or academic degree. Future research using broader samples with diverse age groups and educational backgrounds could provide deeper insight into how these factors influence the relationships among physical activity, creative personality, self-esteem, and smartphone dependence.

Second, the reliance on self-reported measures for exercise, creativity, self-esteem, and smartphone dependence introduces potential biases such as social desirability and recall errors. In particular, exercise time was assessed using a single item adapted from the IPAQ, which, while widely validated, may lack sensitivity in capturing the full variability of exercise behaviors, such as intensity, frequency, and type. Moreover, the single-item format may constrain nuanced interpretation. Similarly, creative personality was measured using an adjective checklist, which may not fully capture the multidimensional nature of creativity as a construct. Finally, smartphone dependence was based solely on self-report, making it susceptible to biases that could influence reported usage patterns. Additionally, the use of a five-point Likert scale without a neutral midpoint may have influenced response patterns. To improve measurement precision, future research should incorporate objective measures or multi-method assessments, such as wearable fitness trackers for exercise and smartphone usage logs, to validate the findings more robustly.

Third, the cross-sectional design of this study limits the ability to infer causal relationships among variables. Longitudinal studies are essential to confirm the temporal sequence of effects and evaluate whether exercise participation leads to sustained improvements in creativity, self-esteem, and reductions in smartphone dependence over time.

Fourth, the study was conducted in Korea, where cultural factors such as collectivism and academic pressures may uniquely influence the dynamics among exercise, psychological traits, and smartphone use. Given that these cultural and educational norms may not be universal, it is important to examine whether similar patterns hold true in other sociocultural settings. Future research should replicate these findings in diverse cultural contexts to assess their generalizability and explore culturally specific pathways that may shape the observed relationships.

Fifth, this study did not include several potential confounding variables—such as academic performance, household socioeconomic status, or screen time for academic purposes—which may influence both exercise participation and smartphone dependence. The absence of these control variables could introduce bias into the observed associations. Future research should incorporate such variables to more rigorously test the hypothesized pathways.

Finally, although creative personality was included as a mediator based on prior research suggesting its potential protective role against behavioral addiction, the direct path from creative personality to smartphone dependence was not statistically significant in this study, with its influence operating entirely through self-esteem. This raises questions about the robustness of creative personality as a stand-alone explanatory factor. Future research should consider revising the theoretical model to reflect this indirect relationship and explore alternative or complementary psychological constructs—such as problem-solving ability, resilience, or self-control—that may more directly account for the mechanisms linking physical activity to smartphone use. Additionally, while this study focused on creative personality and self-esteem, other psychological and contextual factors—including emotional regulation, coping strategies, peer influence, and family support—may also play important roles in adolescent smartphone behavior. A more comprehensive framework, possibly tested through randomized controlled trials (RCTs), could contribute to the development of effective, evidence-based interventions aimed at reducing smartphone dependence and promoting adolescent well-being.

## 5 Conclusion

This study highlights the multifaceted benefits of exercise participation during adolescence, particularly its potential to be associated with reduced smartphone dependence through both direct and indirect psychological pathways. By fostering creative personality and self-esteem, exercise may contribute to healthier developmental outcomes and more balanced technology use among adolescents. These findings underscore the importance of incorporating physical activity into interventions targeting smartphone addiction, especially during adolescence when life-long habits and psychological resilience are formed. Promoting physical activity through educational and community initiatives can serve as a holistic strategy for enhancing adolescent well-being. By addressing both physical and psychological dimensions, such efforts can empower adolescents to build healthier relationships with technology, fostering a more balanced and fulfilling lifestyle.

## Data Availability

Publicly available datasets were analyzed in this study. This data can be found at: https://www.nypi.re.kr/archive/mps.

## Footnotes

^1^ https://www.nypi.re.kr/archive/mps
